# Supplementary material for: Optimal minimal residual disease threshold in pediatric acute myeloid leukemia: A retrospective cohort study based on the TARGET database
Source: PLoS Med. 2026 May 8;23(5):e1005088. doi: 10.1371/journal.pmed.1005088 (PMC13155632; doi:10.1371/journal.pmed.1005088)
Supplement: S1 Code — (ZIP) [file pmed.1005088.s002.zip › S2 code/PROJ8_18_tbl2/PROJ8_18_tbl2_seg2_b.pdf]

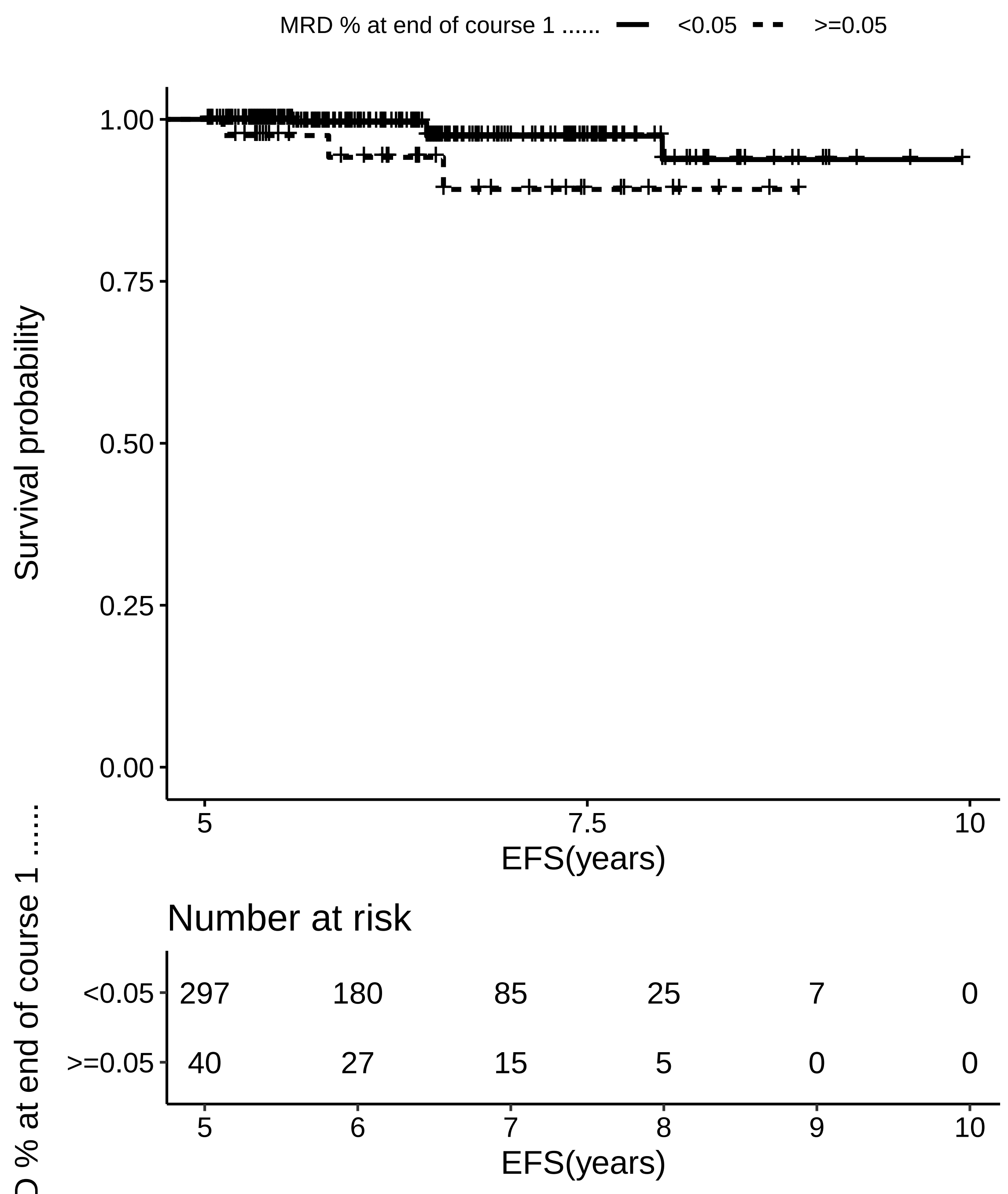

MRD % at end of course 1 ......
<0.05
>=0.05
Survival probability
1.00
0.75
0.50
0.25
0.00
5
7.5
10
EFS(years)
Number at risk
<0.05
>=0.05
297
180
85
25
7
0
40
27
15
5
0
0
5
6
7
8
9
10
EFS(years)
MRD % at end of course 1 ......
